# Supplementary material for: Implementation, feasibility, and acceptability of 99DOTS-based supervision of treatment for drug-susceptible TB in Uganda
Source: PLOS Digit Health. 2023 Jun 30;2(6):e0000138. doi: 10.1371/journal.pdig.0000138 (PMC10313004; doi:10.1371/journal.pdig.0000138)
Supplement: S1 Table — (DOCX) [file pdig.0000138.s001.docx]

**S1 Table: Adherence and Capability, Opportunity, Motivation scores by subgroups (median, IQR)**

|  | **All participants using 99DOTS (N=462)** | **Surveyed participants**  **(N=83)** | | |
| --- | --- | --- | --- | --- |
|  | **Overall adherence** | **Capability^** | **Opportunity^** | **Motivation^** |
| **Gender** |  |  |  |  |
| Men | 57.1% (38.2-75.0) | 4.3 (4.0-4.7) | 4.1 (3.8-4.6) | 4.6 (4.2-4.9) |
| Women | 61.9% (40.7-76.2) | 4.5 (4.3-4.8) | 4.2 (3.8-4.6) | 4.8 (4.4-4.9) |
| **HIV status** |  |  |  |  |
| Positive | 50.6% (52.7-70.8) | 4.3 (4.1-4.7) | 4.1 (3.8-4.4) | 4.7 (4.3-4.9) |
| Negative | 63.7% (43.5-78.6) | 4.5 (4.2-4.8) | 4.2 (3.8-4.7) | 4.7 (4.3-4.9) |
| **Age (years)*** |  |  |  |  |
| Quartile 1 | 57.1% (40.5-73.2) | 4.3 (3.-4.5) | 3.9 (3.5-4.6) | 4.4 (4.2-4.8) |
| Quartile 2 | 54.2% (37.5-71.4) | 4.5 (4.3-4.8) | 4.2 (4.0-4.4) | 4.8 (4.4-4.9) |
| Quartile 3 | 61.9% (43.5-78.6) | 4.3 (4.2-4.7) | 4.2 (3.7-4.6) | 4.6 (4.3-4.9) |
| Quartile 4 | 60.1% (30.1-75.6) | 4.5 (4.2-4.8) | 4.3 (4.0-4.7) | 4.6 (4.5-4.9) |

IQR: Inter-quartile range

* Age quartiles were 18-28, 29-36, 37-48 and 49-89 among the 462 people with TB using 99DOTS and 19-26, 27-35, 36-45 and 48-70 among the 83 people with TB who completed surveys.

^ Capability, opportunity, and motivation were measured using mean response to questions within each category using a Likert scale (1: strongly disagree, 2: disagree, 3: neither, 4: agree, 5: strongly agree), with agreement indicating favorable view of 99DOTS-based TB treatment supervision.
