# Supplementary material for: Implementation, feasibility, and acceptability of 99DOTS-based supervision of treatment for drug-susceptible TB in Uganda
Source: PLOS Digit Health. 2023 Jun 30;2(6):e0000138. doi: 10.1371/journal.pdig.0000138 (PMC10313004; doi:10.1371/journal.pdig.0000138)
Supplement: S2 Table — (DOCX) [file pdig.0000138.s002.docx]

**S2 Table. Demographic and clinical characteristics of people with TB surveyed and not surveyed**

| **Characteristic** | **All participants**  **(N=462)**  **n (%)** | **Surveyed**  **(N=83)**  **n (%)** | **Non-surveyed**  **(N=379)**  **n (%)** | **p-value** |
| --- | --- | --- | --- | --- |
| **Female** | 166 (35.9) | 36 (43.4) | 130 (34.3) | 0.12 |
| **Age** (mean, SD) | 38.9 (0.66) | 37.6 (1.56) | 39.2 (0.73) | 0.36 |
| **Bacteriologically-confirmed TB** | 276 (59.7) | 54 (65.1) | 222 (58.6) | 0.28 |
| **HIV-positive**  **On ART** | 191 (41.3)  191 (100) | 28 (33.7)  28 (100) | 163 (43.0)  163 (100) | 0.11  - |
| **TB Classification**  New  Relapse  Failure  LTFU | 422 (91.3)  32 (6.9)  4 (0.9)  4 (0.9) | 78 (94.0)  3 (3.6)  0  2 (2.4) | 344 (90.8)  29 (7.7)  4 (1.1)  2 (0.5) | 0.15 |
| **Health facility level**  Regional referral hospital  General hospital  Health center IV | 104 (22.5)  255 (55.2)  103 (22.3) | 18 (21.7)  43 (51.8)  22 (26.5) | 86 (22.6)  212 (55.9)  81 (21.3) | 0.59 |

TB: tuberculosis; SD: standard deviation; ART: anti-retroviral therapy
