# Supplementary material for: Implementation, feasibility, and acceptability of 99DOTS-based supervision of treatment for drug-susceptible TB in Uganda
Source: PLOS Digit Health. 2023 Jun 30;2(6):e0000138. doi: 10.1371/journal.pdig.0000138 (PMC10313004; doi:10.1371/journal.pdig.0000138)
Supplement: S3 Table — (DOCX) [file pdig.0000138.s003.docx]

**S3 Table. Survey questions – People with TB**

| **TDF domain** | **Statement** |
| --- | --- |
| **Capability** | |
| Knowledge | - I know how to use DAT - I received adequate training to know how to use 99DOTS - I always know which pill to take next - I know where to find my health care worker’s contact information |
| Memory, attention, and decision processes | - I sometimes forget to call 99DOTs after taking my TB medicines - The reminders I receive on my phone help me to remember to take my TB medicines |
| **Opportunity** | |
| Social influences | - I am worried that using DAT makes it more likely others will find out I have TB - I am comfortable using DAT in front of other people - I am comfortable using DAT outside of my home - I will recommend using DAT to my family or friends if they have TB - DAT helps me feel more connected to my health workers |
| Environmental context and resources | - It is easy for me to access a phone to make daily phone calls to 99DOTS - It takes me too much time to take my TB medicines and call DAT every day - I have had trouble making daily phone calls to 99DOTS because my phone had no charge - I have had trouble making daily phone calls to 99DOTS because of poor network connection - I make fewer trips to the health clinic for my TB treatment because I am using DAT |
| **Motivation** | |
| Optimism | - Using DAT will help me to complete my TB treatment - Using DAT while taking TB medicines will help me get healthy |
| Reinforcement | - The DAT packaging makes it easy for me to remember what to do - My health worker contacts me when I forget to take TB medicines or call 99DOTS - The images inside the 99DOTS envelope make me want to take my TB medicines |
| Intentions | - I intend to call the number revealed after taking TB medicines each day |
| Emotion | - I am concerned about the privacy of my health information collected by the DAT system - The image on the front of the 99DOTS pill pack helps hide that it contains TB medicines - I look forward to hearing the messages played when I call 99DOTS after taking my medicines |

TB: tuberculosis; TDF: Theoretical Domains Framework; DAT: digital adherence technology
