# Supplementary material for: Implementation, feasibility, and acceptability of 99DOTS-based supervision of treatment for drug-susceptible TB in Uganda
Source: PLOS Digit Health. 2023 Jun 30;2(6):e0000138. doi: 10.1371/journal.pdig.0000138 (PMC10313004; doi:10.1371/journal.pdig.0000138)
Supplement: S4 Table — (DOCX) [file pdig.0000138.s004.docx]

**S4 Table. Survey questions – Health workers**

| **TDF domain** | **Statement** |
| --- | --- |
| **Capability** | |
| Knowledge | - I understand the 99DOTS system and how it works - I received adequate training to use 99DOTS |
| Memory, attention and decision processes | - I remember to check the adherence data available in 99DOTS when patients come in for refill visits - The reminders I receive from 99DOTS help me to remember to check on patients who are not taking their medicines |
| Behavior regulation | - The task list in the 99DOTS app helps me manage what I need to do |
| Effort Expectancy* | - It is easy for me to explain how to use 99DOTS to my patients - It is easy for me to enroll a patient on 99DOTS - It is easy for me to identify which patients are not taking their TB medicine using 99DOTS |
| **Opportunity** | |
| Professional role* | - Using 99DOTS to monitor patient adherence is part of my job |
| Social influence | - My coworkers think we should use 99DOTS to monitor patients - My coworkers like using 99DOTS to monitor patients - My patients like using 99DOTS |
| Effort Expectancy* | - Learning to use 99DOTS is easy for me - It is easy for me to access a phone or computer when I need to use 99DOTS - It is easy for me or my coworkers to contact patients who have not taken their TB medicines - Using 99DOTS helps to reduce my workload |
| **Motivation** | |
| Belief about capabilities | - I am confident that I can use 99DOTS to monitor my patients’ adherence to TB treatment |
| Belief about consequences | - Using 99DOTS improves the care I provide to my patients - 99DOTS adherence data helps me provide better support and counseling to my patients |
| Reinforcement | - The images inside the 99DOTS envelope help me educate my patients about TB |
| Risk perception* | - I believe 99DOTS keeps patient information secure |
| Trust factor* | - I believe that 99DOTS data accurately reflects if my patients took their TB medicines or not - I trust that 99DOTS-based TB treatment supervision is safe for my patients |
| Purchase intention* | - I would recommend using 99DOTS to my patients |

*Domain added from Unified Theory of Acceptance and Use of Technology

TB: tuberculosis; TDF: Theoretical Domains Framework; DAT: digital adherence technology
