# Supplementary figures and images for: Implementation, feasibility, and acceptability of 99DOTS-based supervision of treatment for drug-susceptible TB in Uganda
Source: PLOS Digit Health. 2023 Jun 30;2(6):e0000138. doi: 10.1371/journal.pdig.0000138 (PMC10313004; doi:10.1371/journal.pdig.0000138)

**S1 Figure: Engagement of people with TB with 99DOTS by gender, HIV status, and age**

**A.**

**
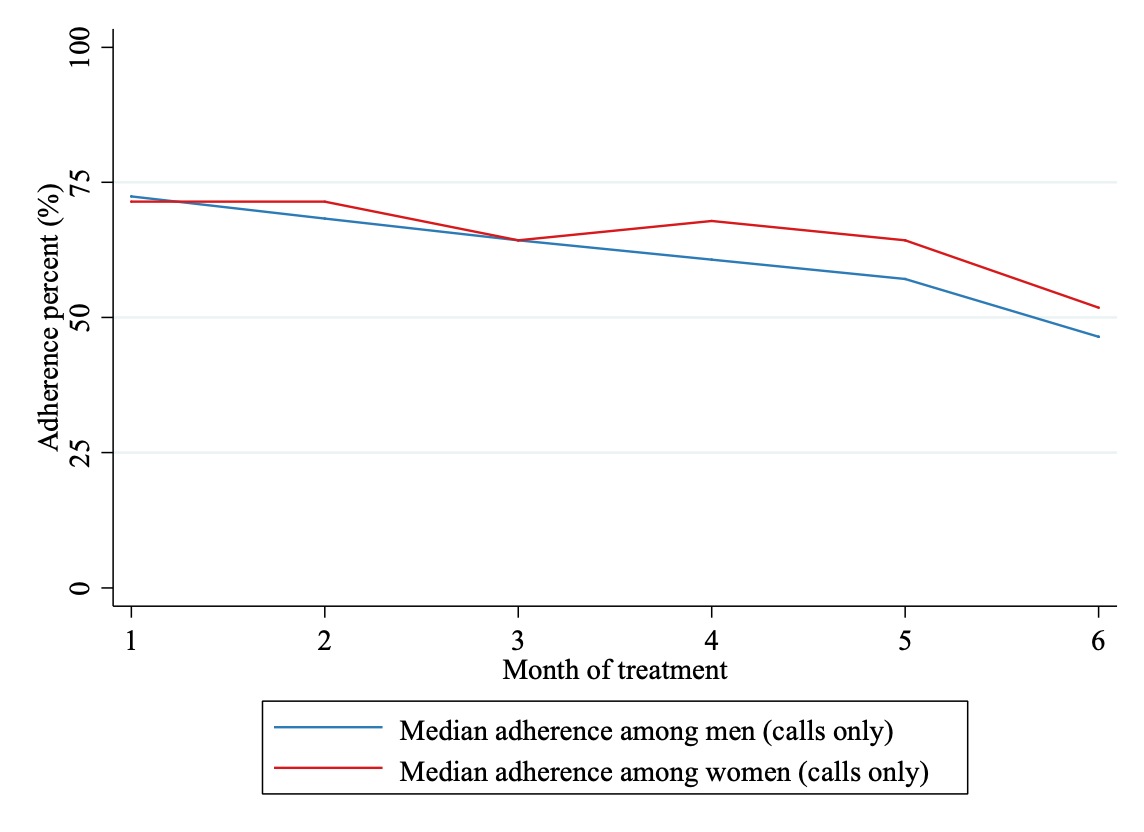
**

**B.**

**
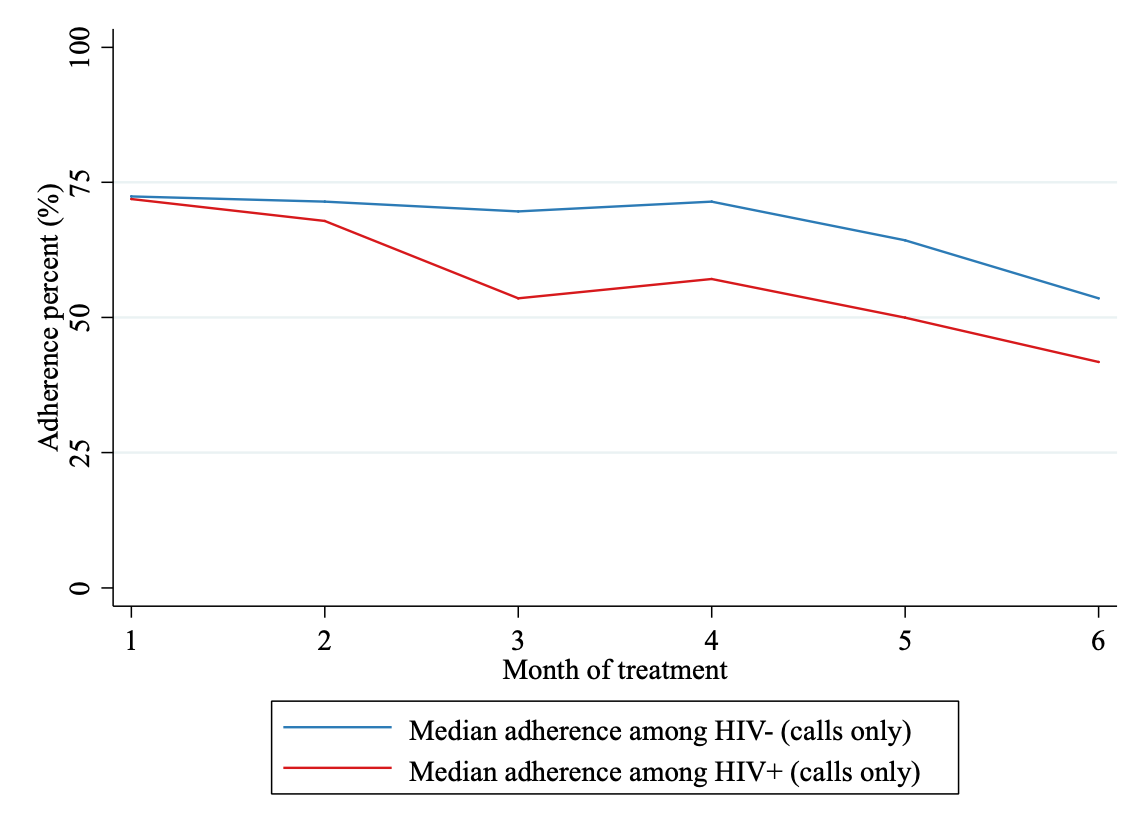
**

**C.**

**
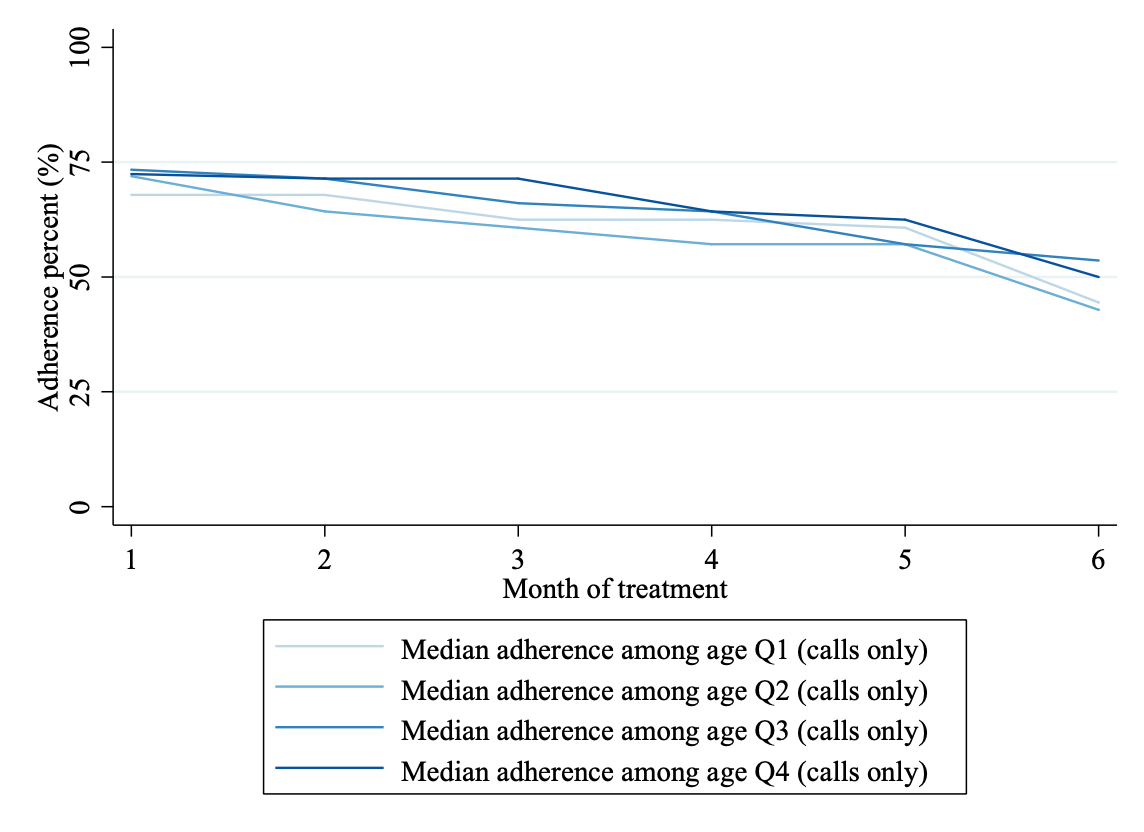
**

Supplement: S1 Fig — Median adherence over months 1–6 of treatment reported by phone call are shown for men and women in panel A, for participants with and without HIV in panel B, and by age quartile (18–28, 29–36, 37–48, and 49–89 years) in panel C. There was no significant difference in trend by age (p = 0.60) or sex (p = 0.41), but people living with HIV had a significantly steeper decline in the proportion of expected doses reported by phone call overs months 1–6 of treatment, compared to people living without HIV (p<0.0001). (DOCX) [file pdig.0000138.s005.docx]

**S2 Figure: Age and opportunity score**

**
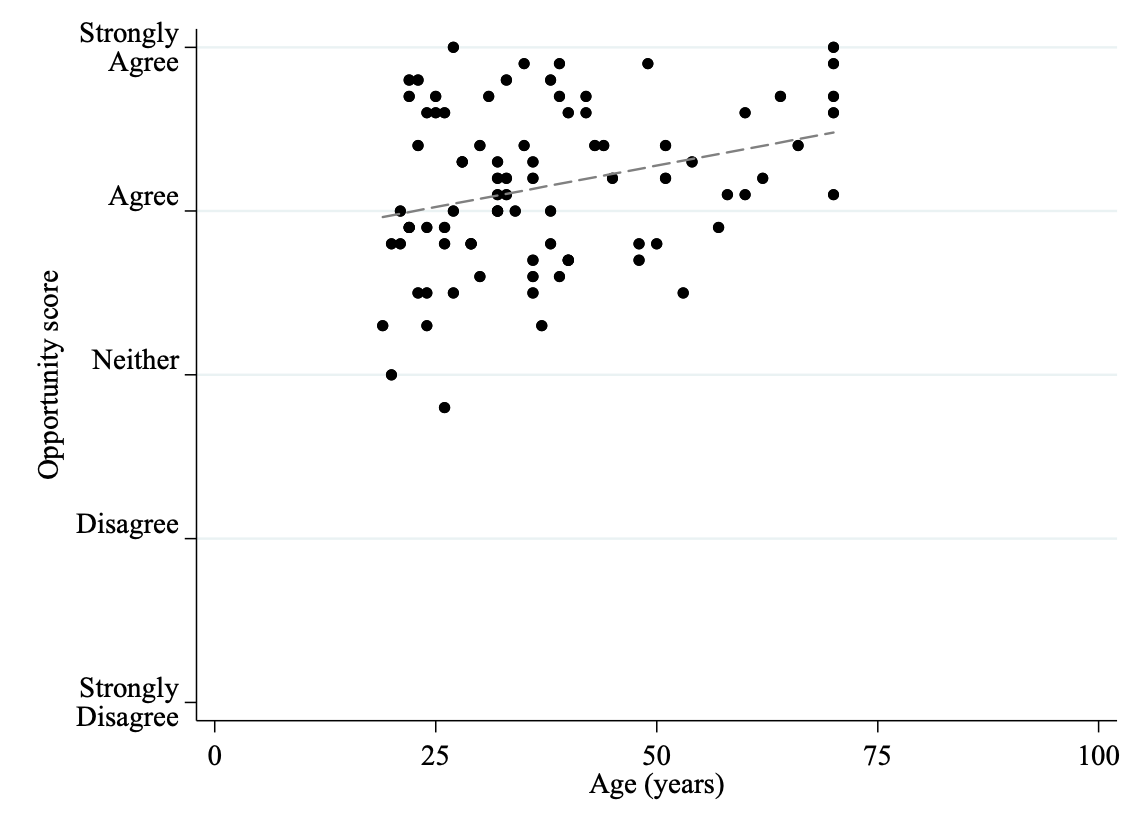
**

Supplement: S2 Fig — Mean opportunity scores were significantly higher among older people with TB using 99DOTS (p = 0.008). (DOCX) [file pdig.0000138.s006.docx]
