# Supplementary material for: Implementation, feasibility, and acceptability of 99DOTS-based supervision of treatment for drug-susceptible TB in Uganda
Source: PLOS Digit Health. 2023 Jun 30;2(6):e0000138. doi: 10.1371/journal.pdig.0000138 (PMC10313004; doi:10.1371/journal.pdig.0000138)
Supplement: Attachment — Submitted filename: review_99DOTS.docx [file pdig.0000138.s011.docx]

*(103-105) The median (..) month of treatment*

1. There is a decrease of 10% of people with TB that use 99DOTS (456 month 1 vs 412 month 6). Could you describe the reasons for discontinuation?

*(106-107) When doses confirmed (..) month of treatment*

Based on the median proportion of expected doses self-reported (103), over 40% of doses were manually added by health workers. This relates to 0,4* 168 = 64 dose days.

2. Did you explore when those doses where confirmed by health workers? Did health workers call each patient on 64 different days to confirm their dose taking on that particular day? Or was batch updating of manual doses done (multiple dose days in once update)? It would be interesting to know the time difference between manual dose submitted by HW vs the actual day that that dose should have happened.
3. To what extent have you assessed whether confirming doses (that should have actually be self-reported by patients) resulted in additional burden for HW?

*(110-112) However, people living with HIV (..) over the course of treatment*

4. To what extent did you explore reasons why people living with HIV report fewer doses and had a greater decline in engagement?

*(118-112) Of 65,045 days (..) people with TB*

5. The number of messages sent is different between row 118 and table 2.
6. On average, how many SMS reminder messages were sent per patient?
7. It looks that more reminder SMS messages were sent to patients than self-reported doses by these same patients. Did you explore to what extent sending reminder messages increase engagement?

*(131-132) Of 409 (88.5%) people with TB who 131 did not call TFNs to report >3 consecutive doses at least once, 261 (63.8%) were documented to have received at least one type of support action*

8. How was this assessed? Did you explore whether when this support action happened, e.g. directly after missing 3 consecutive doses? Or whether people on TB with >3 consecutive doses had at least one support action documented over their course of treatment? The first would be most interested as a measure of fidelity.
9. To what extent do you expect over- or reporting of the support actions? Did you see notable differences between the 18 facilities, where for instance 1 facility contributed much more to the support actions than others?
10. Last, on row (130) 219 extra facility visits is mentioned. How did you differentiate between a regular refill visit and a extra facility visit?

*(217-218) Some people with TB reported stigma and privacy concerns, as well as technical issues with using 99DOTS (phone charging and poor network connection).*

11. According to (180-181) 47/48% of participants struggled with phone charging and poor network connections. Suggest to rephrase “*some*”

*(218-219) However, our data largely represent a more favorable view of 99DOTS than in previous studies. Also on (256-257)*

12. On rows (222-225) engagement and decreased engagement with 99DOTS in this study is similar compared to other studies in India and the 10 other implementation projects. Unclear if the human centered design methods had any influence on the engagement?
13. On rows (250-253) it is mentioned that only 52% of eligible people with TB were enrolled largely due to lack of phone access. This continues to be a major limitation of the use of 99DOTS.
14. To what other studies are you comparing the feasibility and acceptability to justify the statement that your data represent a more favorable view?

*(228-230) 7) Although research is needed to identify opportunities to further increase engagement with 99DOTS, the real-time and quantitative dosing history provided by the platform has strong potential to enhance TB care relative to routine care.*

15. In 40% of the days, the dose was not reported real time, but by a health worker (either that same day or later). Suggest to reword “real-time”
16. This study has a clear focus on human centered design. However, engagement with 99DOTS on a daily basis continues to be relatively low and decreases of time. What other approaches could still be considered to increase daily engagement? Or should 99DOTS and/or any active self-report adherence technology be used at a less frequent basis?
    - In the Philippines, we found that on multiple occasions self reporting was done using unknown phone-numbers (which could be due to change of SIM not updated on Everwell Hub). It might be interesting to see how many "unlinked' self reporting calls have happened in your study.

*(263-264) But overall, our findings support that 99DOTS-based treatment supervision should be offered as an alternative to DOT.*

17. Considering the phone access limitations and daily (low) engagement, some disclaimer might need to be added to this conclusion such as: “*for those people with TB that have access to a mobile phone and are at low risk*”
