# Supplementary material for: Implementation, feasibility, and acceptability of 99DOTS-based supervision of treatment for drug-susceptible TB in Uganda
Source: PLOS Digit Health. 2023 Jun 30;2(6):e0000138. doi: 10.1371/journal.pdig.0000138 (PMC10313004; doi:10.1371/journal.pdig.0000138)
Supplement: Attachment — Submitted filename: Response to reviewers_2023.03.07.docx [file pdig.0000138.s012.docx]

**Response to reviewers**

**PLOS Digital Health Manuscript Number PDIG-D-22-00294R1**

Dear Editorial Board,

Thank you for the opportunity to submit a revised draft of our manuscript entitled “Implementation, feasibility, and acceptability of 99DOTS-based supervision of treatment for drug-susceptible TB in Uganda.” We appreciate the reviewers’ detailed comments. They have helped us to substantially improve the manuscript. Please see below, in blue, our responses to the reviewer’s comments.

**Reviewer #1 (PDF comments)**

• If calls made to 99DOTS were toll free, what is the impact of patients not having airtime to call 99DOTS (table 1). This should be clarified

**Author response**: We set up the toll-free calls for 99DOTS such that they could be made even when the patient had no prepaid airtime on the mobile phone. We have added the following sentence on page 7, “Access to airtime was not needed to make calls to 99DOTS.”

• Table 1 appears before Table 2

**Author response**: We have renumbered the tables, so that Table 1 appears first, followed by Table 2.

• Table 1: is the method to assess adherence done independently of the other options? Example: for patients that 99DOTS is used to assess their adherence to TB medicines, are other methods also used, or is only method exclusively used for this. If mixed methods where used, it is possible that some patients may never have reported adherence using the technology, thus it is unclear what proportion of the patients are only using 99DOTs as part of the study. This section should be clarified.

**Author response**: 99DOTS was the only method used to assess adherence. Adherence was reflected either by patient phone calls to report dosing, or by health workers recording doses manually in the 99DOTS platform (e.g., if health workers called a patient who did not make a phone call and confirmed that the patient had taken their dose). To clarify this, we changed the title of Figure 1 to “Adherence by month of TB treatment, reported by calls to 99DOTS (solid line) or a combination of calls and doses added manually by health workers (dashed line).”

• Line 213: Feasibility is a function of the success of 99DOTs and if 57.7% of doses were recorded by the patients calling 99DOTS, this indicates the >98% of expected doses recorded is not a true factor of the use of 99DOT. Begs to question the feasibility.

**Author response**: We define feasibility as the extent to which 99DOTS could be successfully used or carried out in this setting. In the absence of 99DOTS, health workers have no information on dosing in between refill visits. With 99DOTS, over half of expected doses were reported by phone calls, and this enabled health workers to act on this information and reach out to people with TB on days they were unable to call-in and self-report dose ingestion. We believe this reflects that both people with TB and health workers were able to use the intervention, and that 99DOTS helped facilitate adherence monitoring and support.

• Line 16, Line 289: Indicates that it may not have been FDC medication used by the patients in this study - as they mention that they did not know what pill to take next-, however we know that 99DOTs is used with the FDC due to the packaging of the blisters. This is unclear and somewhat contradictory

**Author response**: All included people with TB were taking fixed dose combination medication in blister packs. The authors believe this comment indicates that people with TB may still find it confusing which pill to take next within the blister pack (i.e. whether to move left to right or top to bottom when selecting the next FDC pill) and may not understand that all pills in the pack are the same. 99DOTS will function the same regardless of how pills are selected.

• Fig 2: Can be argued that “intend to call" is not a motivating factor, but a weakness

**Author response**: We asked participants if they agree or disagree (Likert scale 1-5) with the statement “I intend to call the number revealed after taking TB medicines each day”. Having intention does not guarantee a behavior is performed. However, having intention is a pre-cursor to performing a behavior, and therefore we believe should be considered as a motivating factor.

• Line 256. Only the India study is referenced, however there have been several other studies in Africa in the last couple of years that are more comparable to Uganda. No other literature was compared here.

**Author response**: We are aware of 99DOTS implementation projects in Tanzania, Ethiopia, and Namibia, but only the Tanzania project has published results. We have added comparison with the Tanzania project in the discussion:

- “A study of 99DOTS in Tanzania that used urinalysis to confirm adherence measured by 99DOTS found a positive predictive value of 95%.”
- “In surveys following implementation of 99DOTS in a mining region in Tanzania, all health workers reported that the platform helped them provide better care to people with TB, and 97.5% of people with TB felt 99DOTS helped them adhere and complete treatment. The main challenges they identified were mobile phone access, and unlike in Uganda, a minimum balance was required to call the 99DOTS platform.”
- “We did not find evidence that 99DOTS weakens the relationship between people with TB and health workers; all people with TB agreed that 99DOTS helps them feel more connected to their health workers, with 82% strongly agreeing. This was similar or superior to Tanzania, where 93% of people with TB agreed and 4% strongly agreed that 99DOTS helped them feel more connected to their health worker.”

Added citation:

Efo E, Onjare B, Shilugu L, Levy J, editors. Acceptability, Feasibility and Accuracy of 99DOTS Adherence Technology in Mining Region of Tanzania. 2021 IST-Africa Conference (IST-Africa); 2021 10-14 May 2021.

• Line 272: There is an opportunity here to compare the Uganda study with other studies a where no human centered design of the system was done to see if there is an impact of the design on patient uptake and use.

**Author response**: We appreciate the reviewer’s comment. In the absence of a direct comparison of an HCD-enabled vs. routine 99DOTS platform, we do not feel we can make strong statements about the potential impact of design on patient uptake and use. However, we do suggest the possible positive impact of design in the Discussion:

“A qualitative analysis from India reported high rates of acceptability among healthcare workers and fair acceptability among people with TB. The lower acceptability among people with TB was largely driven by technology concerns (mobile phone access, poor network signal) and stigma.(5) **A minority of people with TB expressed similar concerns in our study, and the overall high scores across COM-B categories among people with TB may in part be due to our extensive customization of the 99DOTS platform using human centered design methods**.(10, 11) We did not find evidence that 99DOTS weakens the relationship between people with TB and health workers; all people with TB agreed that 99DOTS helps them feel more connected to their health workers, with 82% strongly agreeing. This was similar or superior to Tanzania, where 93% of people with TB agreed and 4% strongly agreed that 99DOTS helped them feel more connected to their health worker.(9)”

**Reviewer #2 (word doc comments)**

1.	(103-105) The median (..) month of treatment

There is a decrease of 10% of people with TB that use 99DOTS (456 month 1 vs 412 month 6). Could you describe the reasons for discontinuation?

**Author response**: Of the 462 people included, 50 discontinued use of 99DOTS before month 6. Most people who discontinued use of 99DOTS died during treatment (n=38, 76%), 5 (10%) were lost to follow-up, 4 (8%) failed TB treatment or were diagnosed with drug-resistant TB, and only 3 (6%) completed treatment but discontinued use of 99DOTS.

2.	(106-107) When doses confirmed (..) month of treatment

Based on the median proportion of expected doses self-reported (103), over 40% of doses were manually added by health workers. This relates to 0,4* 168 = 64 dose days. Did you explore when those doses where confirmed by health workers? Did health workers call each patient on 64 different days to confirm their dose taking on that particular day? Or was batch updating of manual doses done (multiple dose days in once update)? It would be interesting to know the time difference between manual dose submitted by HW vs the actual day that that dose should have happened.

**Author response**: Unfortunately, the data available do not allow us to analyze this. From our interactions with health workers, we believe it is common that multiple doses are updated manually at one time. Health workers are prompted to contact patients after three or more doses are missed.

3.	To what extent have you assessed whether confirming doses (that should have actually be self-reported by patients) resulted in additional burden for HW?

**Author response**: In the health worker survey, we asked health workers to agree or disagree with the statement, “Using 99DOTS helps to reduce my workload.” 16 (73%) strongly agreed, 5 (23%) agreed, and 1 (5%) strongly disagreed. With the exception of one health worker, most found that 99DOTS reduced their workload.

4.	(110-112) However, people living with HIV (..) over the course of treatment

To what extent did you explore reasons why people living with HIV report fewer doses and had a greater decline in engagement?

**Author response**: We do not yet know why people living with HIV reported fewer doses overall and had a greater decline in engagement. We are conducting in depth interviews with people with TB, health workers, and TB officers to better understand barriers and facilitators to 99DOTS implementation and will explore differences by HIV status in this ongoing analysis.

5.	(118-112) Of 65,045 days (..) people with TB

The number of messages sent is different between row 118 and table 2.

**Author response**: Thank you for catching this. We have corrected the SMS numbers in the table to match the numbers provided in text.

6.	On average, how many SMS reminder messages were sent per patient?

**Author response**: On average, 137 SMS reminder messages were sent to each person using 99DOTS (standard deviation 44).

7.	It looks that more reminder SMS messages were sent to patients than self-reported doses by these same patients. Did you explore to what extent sending reminder messages increase engagement?

**Author response**: The design of the intervention included a daily SMS reminder. Since all participants on 99DOTS received daily SMS reminders, we are not able to objectively assess the impact of this component. In surveys, we asked people with TB to agree or disagree with the following statement, “The reminders I receive on my phone help me to remember to take my TB medicines.” 54 (65%) people with TB strongly agreed, 24 (29%) agreed, 3 (4%) disagreed, and 2 (2%) strongly disagreed.

8.	(131-132) Of 409 (88.5%) people with TB who 131 did not call TFNs to report >3 consecutive doses at least once, 261 (63.8%) were documented to have received at least one type of support action

How was this assessed? Did you explore whether when this support action happened, e.g. directly after missing 3 consecutive doses? Or whether people on TB with >3 consecutive doses had at least one support action documented over their course of treatment? The first would be most interested as a measure of fidelity.

**Author response**: This was assessed using support actions logged on 99DOTS by health workers. Of all patients who did not call TFNs to report >3 consecutive doses at least once during treatment, we assessed how many had at least one support action (phone call, facility visit, home visit) recorded. We agree it would have been ideal to assess whether the support action occurred directly after missing 3 doses. However, the data available through the 99DOTS platform does not allow for this.

9.	To what extent do you expect over- or reporting of the support actions? Did you see notable differences between the 18 facilities, where for instance 1 facility contributed much more to the support actions than others?

**Author response**: In surveys, we asked people with TB to agree or disagree with the following statement, “My health worker contacts me when I forget to take TB medicines or call 99DOTS.'' 36 (43%) strongly agreed, 24 (29%) agreed, 7 (8%) neither agreed nor disagreed, 5 (6%) disagreed, and 11 (13%) strongly disagreed. We think there was under-reporting of support actions by healthcare workers, but we are not able to quantify to what extent actions were unreported. To log a support action, health workers had to manually log the interaction in the 99DOTS app. We are working with Everwell Health Solutions on better ways to log and track the timing of support actions.

The number of support actions logged per person with TB varied across sites. The average number of phone calls logged per person with TB ranged from 0 at three sites to 7.6 calls per person at one site; the average number of home visits logged per person ranged from 0 at seven sites to 1.2 home visits per person at one site. Facility visits ranged from 0 at five sites to 2.6 facility visits per person at one site.

10.	Last, on row (130) 219 extra facility visits is mentioned. How did you differentiate between a regular refill visit and a extra facility visit?

**Author response:** The 99DOTS App was designed to capture refill and facility visits separately. Providers were trained to document “refill visit” if the patient made a scheduled visit to the facility to pick more drugs and to enter “facility visit” if a patient visited the facility for any other reason other than refill of drugs.

11.	(217-218) Some people with TB reported stigma and privacy concerns, as well as technical issues with using 99DOTS (phone charging and poor network connection).

According to (180-181) 47/48% of participants struggled with phone charging and poor network connections. Suggest to rephrase “some”

**Author response**: Thank you for this suggestion. We have edited this line to read, “However, some struggled with phone charging (n=39, 47%) and poor network connections (n=40, 48%).”

12.	(218-219) However, our data largely represent a more favorable view of 99DOTS than in previous studies. Also on (256-257)

On rows (222-225) engagement and decreased engagement with 99DOTS in this study is similar compared to other studies in India and the 10 other implementation projects. Unclear if the human centered design methods had any influence on the engagement?

**Author response**: We do not have evidence that the use of human centered design had direct influence on patient engagement with 99DOTS; this is certainly an area for further human centered design work. However, we believe the design methods may have improved the acceptability of 99DOTS in Uganda. In the survey, we asked people with TB about certain aspects of the intervention that were developed through human centered design:

- The images inside the 99DOTS envelope make me want to take my TB medicines: mean score 4.7 (95% CI 4.6, 4.8)
- The DAT packaging makes it easy for me to remember what to do: 4.8 (95% CI 4.7, 4.8)
- I know where to find my health care worker’s contact information: 4.4 (95% CI 4.2, 4.6)

The messages played back to the caller after calling to log a dose with 99DOTS were developed through human-centered design to incorporate motivational messaging. Three survey questions asked about this aspect of the intervention and its intended motivations:

- I look forward to hearing the messages played when I call 99DOTS after taking my medicines: 4.8 (95% CI 4.7, 4.9)
- Using DAT will help me to complete my TB treatment: 4.9 (95% CI 4.8, 4.9)
- Using DAT while taking TB medicines will help me get healthy: 4.9 (95% CI 4.8, 4.9)

We note in the Discussion that: “A qualitative analysis from India reported high rates of acceptability among healthcare workers and fair acceptability among people with TB. The lower acceptability among people with TB was largely driven by technology concerns (mobile phone access, poor network signal) and stigma.(5) A minority of people with TB expressed similar concerns in our study, and the overall high scores across COM-B categories among people with TB may in part be due to our extensive customization of the 99DOTS platform using human centered design methods.(10, 11)”

13.	On rows (250-253) it is mentioned that only 52% of eligible people with TB were enrolled largely due to lack of phone access. This continues to be a major limitation of the use of 99DOTS.

**Author response**: We thank the reviewer for this comment. We agree that lack of access to phones remains a major limitation to the use of 99DOTS. In the next phase of 99DOTS implementation following the trial, we included an option of providing low-cost mobile phones to people with TB that did not have access to a phone. In this subsequent period, >85% of people with TB have been enrolled on 99DOTS (manuscript in preparation).

14.	To what other studies are you comparing the feasibility and acceptability to justify the statement that your data represent a more favorable view?

**Author response**: The experience in Uganda was largely more favorable than the experience reported in India. We have clarified in text:

“However, our data largely represent a more favorable view of 99DOTS than presented from India, where it was reported to increased workload for health workers and reduce interaction between people with TB and health workers.(3) Acceptance of 99DOTS by people with TB in India was hindered by perceptions of reduced contact with health workers and poor counseling, and health workers reported poor facilitating conditions.(5)”

We have also added additional comparison with the Tanzania experience, as explained in our response to Reviewer 1.

15.	(228-230) 7) Although research is needed to identify opportunities to further increase engagement with 99DOTS, the real-time and quantitative dosing history provided by the platform has strong potential to enhance TB care relative to routine care.

In 40% of the days, the dose was not reported real time, but by a health worker (either that same day or later). Suggest to reword “real-time”

**Author response**: We acknowledge that patients did not report all doses via phone call; however, in comparison to standard community-based DOT, which provides no information to health workers between refill visits, 99DOTS provides some real-time dosing information to health workers every day. We defer to the editor on whether this language is appropriate.

16.	This study has a clear focus on human centered design. However, engagement with 99DOTS on a daily basis continues to be relatively low and decreases of time. What other approaches could still be considered to increase daily engagement? Or should 99DOTS and/or any active self-report adherence technology be used at a less frequent basis? In the Philippines, we found that on multiple occasions self reporting was done using unknown phone-numbers (which could be due to change of SIM not updated on Everwell Hub). It might be interesting to see how many "unlinked' self reporting calls have happened in your study.

**Author response**: We agree that sustained engagement is a major challenge for 99DOTS. In our next iteration of the project, we have task shifted adherence monitoring to community health workers, with the hope that they will provide more prompt attention and adherence support follow-up to people who do not self-report dosing. But ultimately, technologies that do not require people to actively report adherence may be needed or even with such technologies, engagement may decrease over time. Even if the latter were true, we believe some dosing information is better than none (as is the case currently) and enables health workers to follow-up with people who may be having adherence challenges.

17.	(263-264) But overall, our findings support that 99DOTS-based treatment supervision should be offered as an alternative to DOT.

Considering the phone access limitations and daily (low) engagement, some disclaimer might need to be added to this conclusion such as: “for those people with TB that have access to a mobile phone and are at low risk”

**Author response**: We agree that a phone is needed to use 99DOTS, but provision of a phone may increase uptake (as we are seeing in our subsequent 99DOTS scale-up project). In comparison to standard community-based DOT, which provides no information to health workers between refill visits, we would still argue that 99DOTS enables closer monitoring. Based on the reviewer’s comment, we modified the conclusion to:

“But overall, our findings support that 99DOTS-based treatment supervision should be offered as an alternative to DOT for people with TB who have access to a phone.”
