# Supplementary material for: Implementation, feasibility, and acceptability of 99DOTS-based supervision of treatment for drug-susceptible TB in Uganda
Source: PLOS Digit Health. 2023 Jun 30;2(6):e0000138. doi: 10.1371/journal.pdig.0000138 (PMC10313004; doi:10.1371/journal.pdig.0000138)
Supplement: Attachment — Submitted filename: Reviewer Comments.pdf [file pdig.0000138.s010.pdf]

Reviewer Comments

- If calls made to 99DOTS were toll free, what is the impact of patients not having airtime to call 99DOTS (table 1). This should be clarified

- Table 1 appears before Table 2

- Table 1: is the method to assess adherence done independently of the other options? Example: for patients that 99DOTS is used to assess their adherence to TB medicines, are other methods also used, or is only method exclusively used for this. If mixed methods where used, it is possible that some patients may never have reported adherence using the technology, thus it is unclear what proportion of the patients are only using 99DOTs as part of the study. This section should be clarified.

- Line 213: Feasibility is a function of the success of 99DOTs and if 57.7% of doses were recorded by the patients calling 99DOTS, this indicates the >98% of expected doses recorded is not a true factor of the use of 99DOT. Begs to question the feasibility.

- Line 16, Line 289: Indicates that it may not have been FDC medication used by the patients in this study - as they mention that they did not know what pill to take next-, however we know that 99DOTs is used with the FDC due to the packaging of the blisters. This is unclear and somewhat contradictory

- Fig 2: Can be argued that “intend to call" is not a motivating factor, but a weakness

- Line 256. Only the India study is referenced, however there have been several other studies in Africa in the last couple of years that are more comparable to Uganda. No other literature was compared here.

- Line 272: There is an opportunity here to compare the Uganda study with other studies a where no human centered design of the system was done to see if there is an impact of the design on patient uptake and use.
